# Experimental and Theoretical Studies of Mo/Au Schottky Contact on Mechanically Exfoliated β-Ga_2_O_3_ Thin Film

**DOI:** 10.1186/s11671-018-2837-2

**Published:** 2019-01-03

**Authors:** Zhuangzhuang Hu, Qian Feng, Zhaoqing Feng, Yuncong Cai, Yixian Shen, Guangshuo Yan, Xiaoli Lu, Chunfu Zhang, Hong Zhou, Jincheng Zhang, Yue Hao

**Affiliations:** State Key Discipline Laboratory of Wide Band Gap Semiconductor Technology, School of Microelectronics, Xidian University, Xi’an, 710071 China

**Keywords:** β-Ga_2_O_3_ Schottky diode, Carrier transport mechanism, Reverse bias, Schottky emission, Breakdown voltage

## Abstract

We studied the reverse current emission mechanism of the Mo/β-Ga_2_O_3_ Schottky barrier diode through the temperature-dependent current-voltage (I-V) characteristics from 298 to 423 K. The variation of reverse current with the electric field indicates that the Schottky emission is the dominant carrier transport mechanism under reverse bias rather than the Frenkel–Poole trap-assisted emission model. Moreover, a breakdown voltage of 300 V was obtained in Fluorinert ambient with an average electric field of 3 MV/cm in Mo/β-Ga_2_O_3_ Schottky barrier diode. The effects of the surface states, on the electric field distribution, were also analyzed by TCAD simulation. With the negative surface charge densities increasing, the peak electric field reduces monotonously. Furthermore, the Schottky barrier height inhomogeneity under forward bias was also discussed.

## Background

Recently, the ultra-wide bandgap semiconductor β-Ga_2_O_3_ has attracted lots of interests for its excellent characteristics, such as high chemical stability, large direct wide band gap of 4.8–4.9 eV, high theoretical breakdown electric field (*E*_BR_) of 8 MV/cm, and high Baliga’s figure-of-merit of 3400, which is about ten times larger than that of SiC and four times larger than that of GaN [1–3]. The combination of all these properties with the high quality, large area, and cost-effective β-Ga_2_O_3_ substrate grown by melt growth techniques makes β-Ga_2_O_3_ a preferred material for high-voltage and high-power electronics applications [4–9]. As a promising electronic device, β-Ga_2_O_3_ Schottky barrier diodes (SBD) were fabricated with various anode electrode metals, including Cu [8], Pd [10], Pt [5, 6, 11–13], Au [10, 14], Ni [13, 15–18], and TiN [12], and its forward and reverse electrical characteristics, such as the specific on-resistance, *I*_on_/*I*_off_ ratio, barrier heights, reverse leakage current, and breakdown voltage, were comprehensively investigated. The inhomogeneous Schottky barrier height and non-saturating reverse bias current were reported in β-Ga_2_O_3_ SBDs [6, 8, 11, 18, 19] while much less information was known about carrier transport mechanism under reverse bias, which is essential for the breakdown voltage enhancement.

In addition, there is no investigation that has been made to analyze the emission mechanisms of Mo/β-Ga_2_O_3_ contact. If there are some traps or defects in the β-Ga_2_O_3_ substrate, the leakage current will be found to be in agreement with the Frenkel–Poole emission model, and the reverse current is the emission of electrons from a trapped state near the metal-semiconductor interface. Otherwise, the main process in reverse current will be dominated by the Schottky emission that the electrons over the Schottky barrier result in a reverse current. β-Ga_2_O_3_ crystal also has one unique property, a large lattice constant of 12.23 Å along [100] direction, which allows a facile cleavage into thin belts or nano-membranes [9, 20]. So in this work, we mechanically exfoliated large-scale β-Ga_2_O_3_ from low dislocation density bulk substrate, and for the first time, the thermally stable Molybdenum (Mo) was chosen as the anode electrode metal to fabricate the β-Ga_2_O_3_ vertical Schottky barrier diodes. The electrical conduction mechanism under the reverse bias was discussed at the temperature range from 298 to 423 K. This work provides insights into carrier transport mechanisms that can help improve functionalities of β-Ga_2_O_3_-based devices.

## Methods/Experimental

**Fig. 1:**
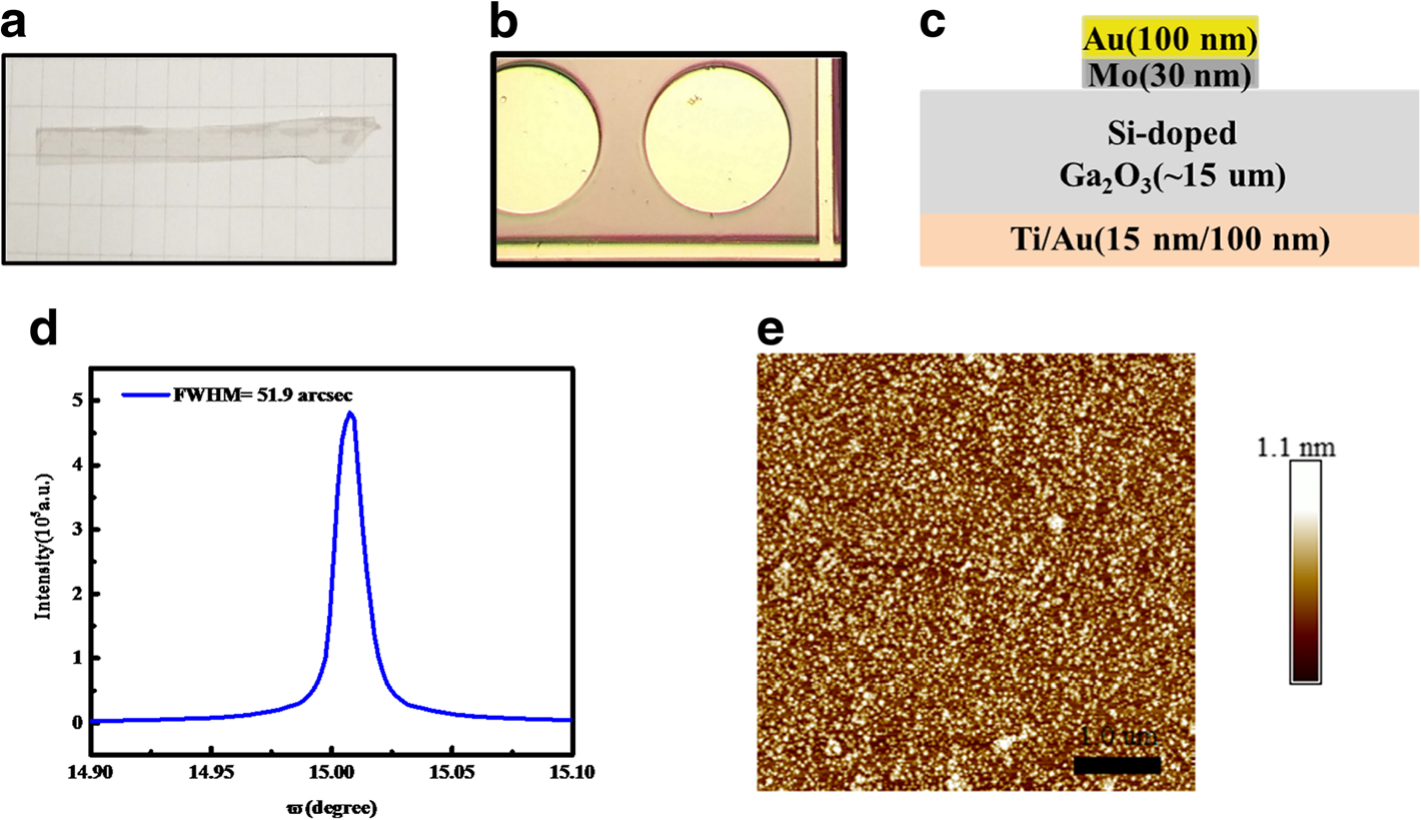
**a** The Sn-doped β-Ga_2_O_3_ substrate with the thickness of 300 μm. **b** The Schottky anode electrodes formed on the front side with diameters of 100 μm. **c** The structure of the schematic across section of the β-Ga_2_O_3_ SBD. **d** XRD rocking curve and **e** AFM image of the β-Ga_2_O_3_ drift layer mechanically exfoliated from (100) β-Ga_2_O_3_ substrate

As shown in Fig. 1a, b, the Schottky barrier diode was fabricated on the β-Ga_2_O_3_ (100) film mechanically exfoliated from the Sn-doped β-Ga_2_O_3_ substrate, with the thickness of 15 μm and electron concentration of 2 × 10^17^ cm^− 3^. As presented in Fig. 1d, e, the full width at half-maximum (FWHM) and root mean square (RMS) were estimated to be 51.9 arcsec and 0.19 nm, respectively, by high resolution X-ray diffraction (HRXRD) and atomic force microscope (AFM) measurements. Excellent crystal quality and smooth surface were confirmed by the measurement. After wet chemical cleaning, the Ti/Au (20 nm/100 nm) metal stack was deposited using E-beam evaporation on the back side and followed by the rapid thermal annealing (RTA) at 600 °C for 60 s under nitrogen atmosphere to form the Ohmic contact. The circular Schottky anode electrodes with diameters of 100 μm were formed on the front side by evaporation of Mo/Au (40 nm/100 nm) metals and lift-off process. Figure 1c shows the structure of the schematic cross section of the β-Ga_2_O_3_ SBD in this work.

## Results and Discussion

**Fig. 2:**
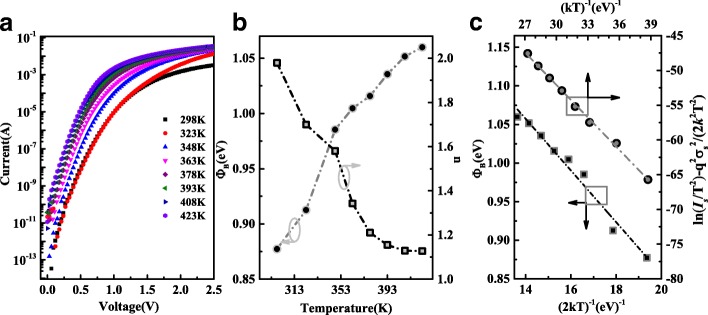
**a** Forward I-V characteristics of Mo/β-Ga_2_O_3_ Schottky barrier diodes at different temperatures. **b** Temperature dependence of the ideality factor and Schottky barrier height of β-Ga_2_O_3_ Schottky barrier diode. **c** Plots of *ϕ*_*ap*_ versus q/2*k*T and modified Richardson plot versus 1/*k*T for the β-Ga_2_O_3_ Schottky barrier diodes

The current-voltage (I-V) characteristics of Au/Mo/β-Ga_2_O_3_ Schottky barrier diodes were investigated using a Keithley 4200 semiconductor characterization system between 298 and 423 K. As shown in Fig. 2a, the *I*_on_/*I*_off_ ratio is close to 10^10^ at 298 K, indicating a good rectifying behavior. For the forward bias from 0.1 to 0.7 V, the semilogarithmic I-V curves are almost liner and display a strong temperature dependence behavior. With the forward bias further increasing, the deviation from linearity of the I-V curves is ascribed to the series resistance of the Schottky barrier diode and the relationship between the applied voltage and the current can be expressed as $I={I}_s\left\{\exp \left[\frac{q\left(V-{IR}_s\right)}{nkT}\right]-1\right\}$ [21–23], where *V* is the applied voltage, *R*_s_ the series resistance, *T* the absolute temperature, *k* the Boltzmann constant, *n* the ideality factor, and *I*_*s*_ is the reverse saturation current. The *n* and *I*_*s*_ can be determined from the slope and intercept of the *ln*I-V plots, respectively. For the ideal Schottky barrier diode, the ideality factor *n* should be equal to unity. The higher the *n*, the greater the deviation from the thermal emission (TE) model. In addition, according to the equation ${\phi}_b=\frac{kT}{q}\ln \left[\frac{AA^{\ast }{T}^2}{I_s}\right]$ [21–23], the values of *ϕ*_*b*_ at different temperatures were also determined, as shown in Fig. 2b, where *ϕ*_*b*_ is the barrier height, *A* is the diode area and A^*^ is the effective Richard constant 40.8 A cm^−2^ K^− 2^ with the β-Ga_2_O_3_ effective mass of m^*^ *=* 0.34 *m*_0_ [5, 24]. With temperature increasing from 298 to 423 K, the *ϕ*_*b*_ increases while *n* decreases, indicating another transport mechanism also contributing to the current transport and leading to the deviation of the I-V characteristics from the pure TE model, which has been reported previously in β-Ga_2_O_3_ Schottky barrier diodes [25] and other wide bandgap devices [26–30]. The barrier height inhomogeneity analysis can be described by a Gaussian distribution in barrier heights,

1 $$ {\phi}_b=\overline{\phi_{b0}}\left(T=0\right)-\frac{q{\sigma}_s^2}{2 kT} $$

The values of mean barrier height $\overline{\phi_{b0}}$ and the standard deviation *σ*_*s*_ are extracted to be 1.55 eV and 0.186 eV, respectively, from Fig. 2c. Furthermore, considering the barrier height inhomogeneities, the conventional Richardson plot is modified as follows:

2 $$ \ln \left(\frac{I_{\mathrm{s}}}{T^2}\right)-\left(\frac{q^2{\sigma}_{\mathrm{s}}^2}{2{k}^2{T}^2}\right)=\ln \left({AA}^{\ast}\right)-\frac{q\overline{\phi_{b0}}}{kT} $$

As shown in Fig. 2c, the modified $\ln \left({I}_{\mathrm{s}}/{T}^2\right)-\left({q}^2{\sigma}_{\mathrm{s}}^2/2{k}^2{T}^2\right)$ versus 1/*kT* is a straight line. The intercept of the curve is used to obtain the A^*^ of 44.7 A cm^−2^ K^−2^, which is very close to the theoretical value of β-Ga_2_O_3_ of 40.8 A cm^−2^ k^−2^. Hence, the barrier inhomogeneities at metal/semiconductor interface for β-Ga_2_O_3_ SBD can be explained by TE with Gaussian distribution of barrier over the SBHs.

**Fig. 3:**
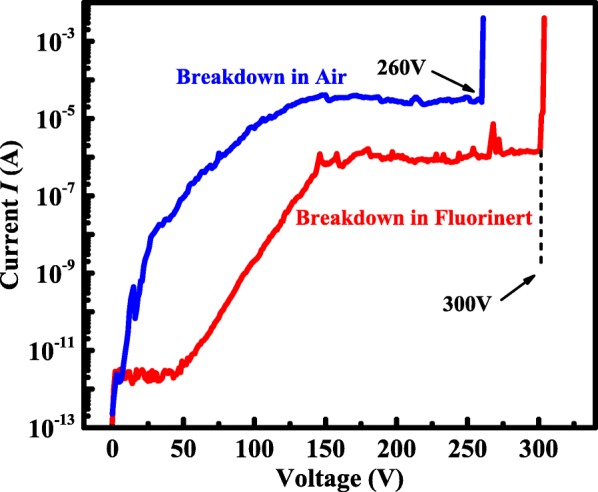
The reverse I-V characteristics of the β-Ga_2_O_3_ samples at room temperature respectively in Fluorinert and air

**Fig. 4:**
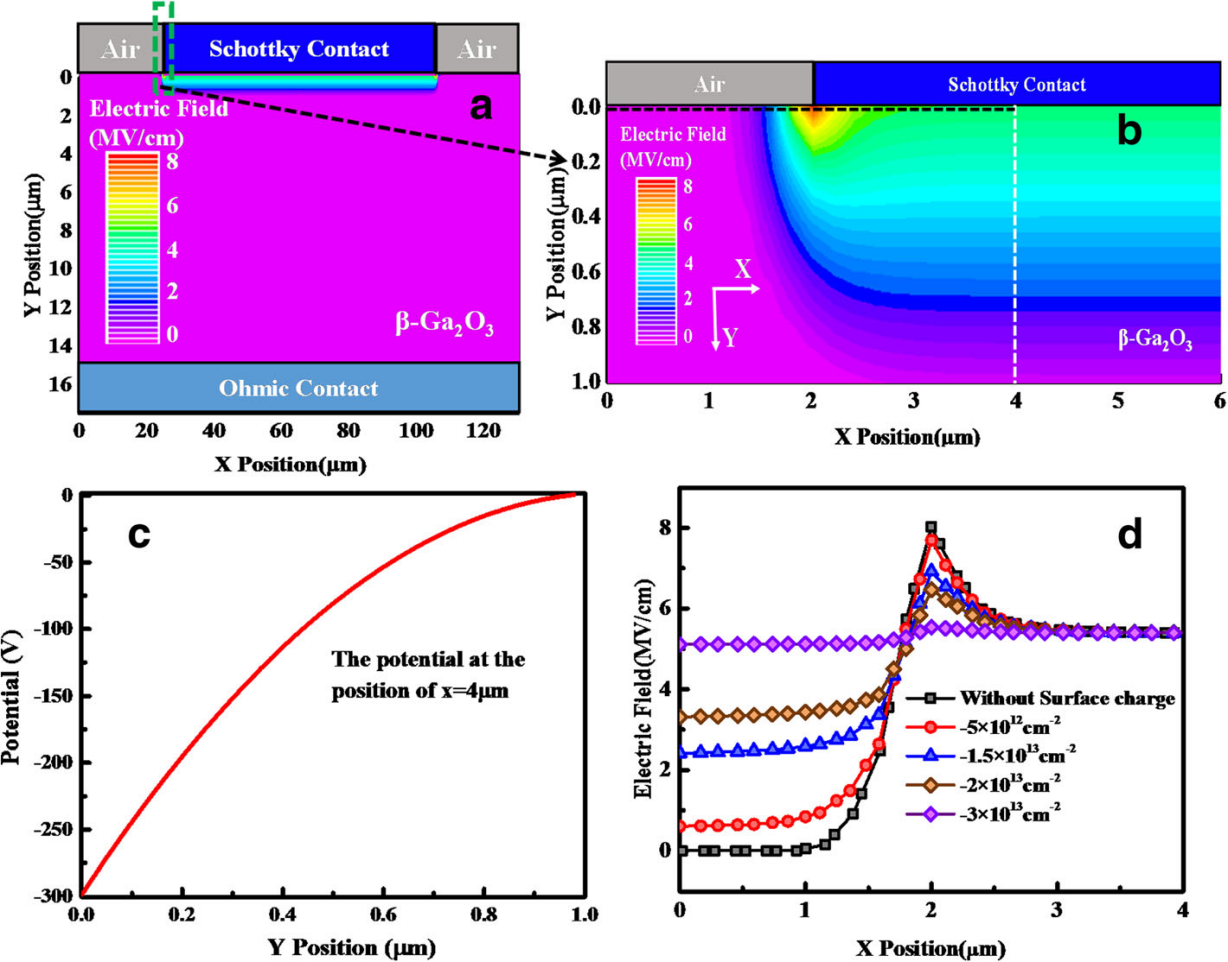
**a** Off-state TCAD electric field simulation of the Schottky barrier diodes under − 300 V bias. **b** The electric field simulation of the selected regions in green dashed box. The potential along the *y* axis at *x* = 4 μm is present in (**c**), and the electric field at the edge of the Schottky contact reduced with different effective negative surface charge densities are present in (**d**)

The room temperature reverse breakdown measurement was also carried out by using Agilent B1505A high-voltage semiconductor analyzer system, as shown in Fig. 3. The breakdown voltage was 260 V while it was 300 V with the sample submerged in Fluorinert ™ produced by 3M company which can prevent air breakdown under high reverse bias. In order to understand the distribution of the electric field, numerical simulation was performed with ATLAS software, as shown in Fig. 4a, b. With the distance increasing from the interface between the semiconductor and the anode to about 1 μm, the electric field gradually decreasing. At the position of *x* = 4 μm, the average electric field is 3 MV/cm, calculated from Fig. 4c. Also shown in Fig. 4d, at the position of *y* = 1 nm, the maximum electric field at breakdown voltage was about 8 MV/cm at the edge of the Schottky contact, which is about 2.7 times that of the average electric field. As reported by A. J. Green et al [31] and K. Zeng et al [32], the peak electric field and the average electric field of the electrode edge were 5.3, 3.8 MV/cm and 6.1, 4.4 MV/cm, respectively, and the peak electric field of Mo/Ga_2_O_3_ Schottky diode is relatively high. It is supposed that the β-Ga_2_O_3_ nano-membrane obtained by mechanical exfoliation has a large number of dangling bonds and surface states which will capture electrons to deplete the carriers from anode to cathode under reverse bias [33]. Taken the negative surface charge into account, the simulation result showed the electric field at the edge of the Schottky contact reduced with negative surface charge densities increasing from 0.5 × 10^13^ cm^−2^ to 3 × 10^13^ cm^−2^, respectively. Especially with the negative surface charge densities of 3 × 10^13^ cm^−2^, the peak electric field at the edge of the Schottky contact is about 5.2 MV/cm. Therefore, the reverse breakdown voltage 300 V can be achieved on the β-Ga_2_O_3_ nano-membrane with *N*_D_ = 3 × 10^17^ cm^−2^ without any edge termination structures. As shown in Fig. 4d,because of the existence of interface state at *X*-position below 2 μm, the electrons can be trapped and the depletion region can be formed, resulting in the electric field in the *Y* direction. As the interface state concentration increases, the electric field in the *Y* direction increases, although the electric field in the *X* direction approaches zero. So the electric field increases at *X*-position below 2 μm.

**Fig. 5:**
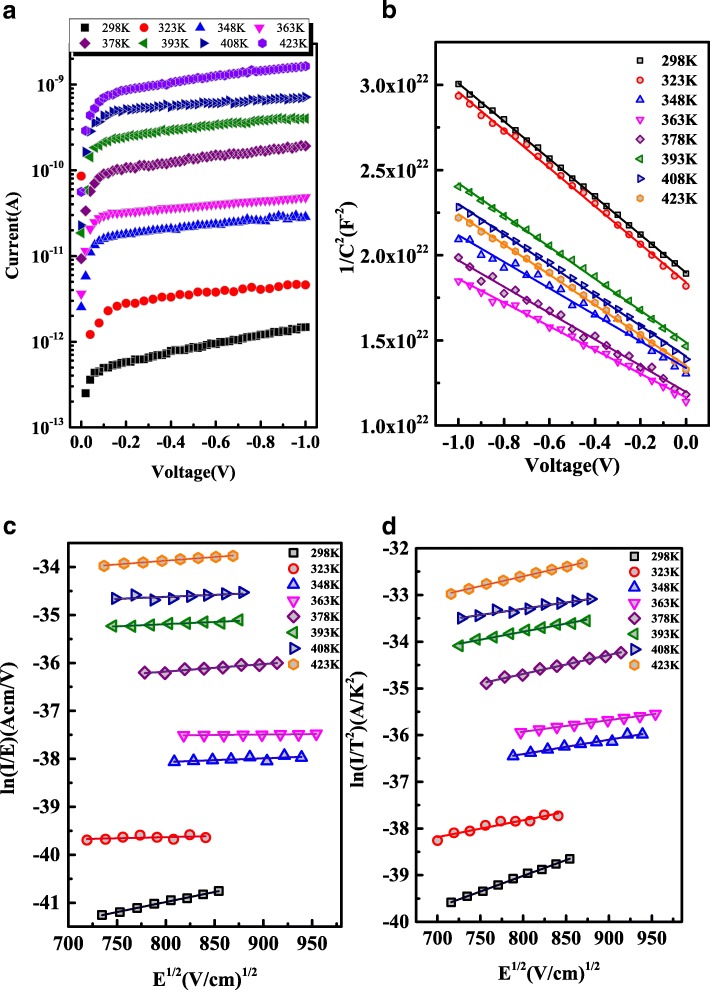
**a** Reverse I-V characteristics of Mo/β-Ga_2_O_3_ Schottky barrier diodes at different temperature. **b** Temperature dependence of 1/C^2^ characteristics of the Mo/ β-Ga_2_O_3_ Schottky barrier diodes. The electric field dependence analysis of Mo/β-Ga_2_O_3_ Schottky contact with different mechanism. **c** Poole–Frenkel mechanism (*I*/*E*) versus *E*^1/2^ and **d** Schottky mechanism *ln*(*I*/*T*^2^) versus *E*^1/2^

On the other hand, with the reverse bias *V*_*re*_ increasing, the leakage current *I*_*re*_ increases rather than saturate for |*V* | > *3 k*_*B*_*T/q*, as shown in Fig. 5a, which is inconsistent with the TE theory. Therefore, the electric-field enhanced thermionic emission was considered to discuss the dependence of the *I*_*re*_ on *V*_*re*_*,* including Poole–Frenkel emission and Schottky emission [34, 35]. In Poole–Frenkel emission, the electrons transport from metal into the semiconductor via a trapped state and the *I*_*re*_ is given by

3 $$ {I}_{re}\propto E\exp \left(\frac{q}{kT}\sqrt{\frac{qE}{{\pi \varepsilon}_S}}\right) $$

while in Schottky emission, the electrons will gain enough energy to overcome the barrier at the metal/semiconductor to form the current and the *I*_*re*_ can be expressed by

4 $$ {I}_{re}\propto {T}^2\exp \left(\frac{q}{2 kT}\sqrt{\frac{qE}{{\pi \varepsilon}_S}}\right) $$

where *ε*_*s*_ is the permittivity of the β-Ga_2_O_3_ (~ 10 ε_0_) and *E* is the applied electric field, calculated by $E\kern0.5em =\kern0.5em \sqrt{\frac{2{qN}_D}{\varepsilon_S}\left(V+{V}_{bi}-\frac{k_BT}{q}\right)}$, *N*_*D*_ is the donor density of the β-Ga_2_O_3_, and *V*_*bi*_ is the built-in potential. As shown in Fig. 5b, *N*_*D*_ and *V*_*bi*_ can be extracted from the slope and the intercept of the inverse square capacitance (1/*C*^*2*^) versus the *V*_*re*_ plots using the following expression

5 $$ \frac{1}{C^2}=\frac{2\left({V}_{\mathrm{bi}}- kT/q-V\right)}{q{\varepsilon}_s{A}^2{N}_D} $$

**Fig. 6:**
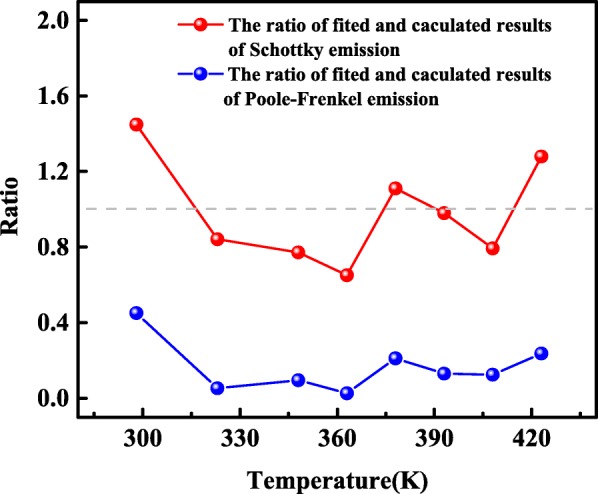
Relative coefficient plots of Poole–Frenkel emission *N*_*PF*_ (=*S*_*m-PF*_/*S*_*PF*_) and Schottky emission *N*_*S*_ (=*S*_*m-S*_/*S*_*S*_) versus temperature

If the curve of *ln*(*I/T*^2^) versus *E*^1/2^ is linear, the Schottky emission mechanism is dominant. And if the plot of *ln*(*I*/*E*) versus *E*^1/2^ is liner, the Poole–Frenkel emission dominates the reverse current transport. Figure 5c, d depicts the plots of *ln*(*I*/*E*) and *ln*(*I*/*T*^2^) versus *E*^1/2^, respectively. Both sets of the curves are linear, indicating not only the Poole–Frenkel emission but also the Schottky emission are present. In order to clarify the dominant carrier transport mechanism, the slope of the curves, or the emission coefficient can be expressed as [34–36].

6 $$ S=\frac{q}{nkT}\sqrt{\frac{q}{\pi \varepsilon}} $$

where *n* = 1 is for the Poole–Frenkel emission (*S*_*PF*_) and *n* = 2 for the Schottky emission (*S*_*S*_). The experimental values of *S* are denoted as *S*_*m-PF*_ and *S*_*m-S*_ for Poole–Frenkel and Schottky emission given by the slope of the curves in Fig. 5c, d, respectively. The ratios of the experimental value to the theoretical value, *N*_*PF*_ (=*S*_*m-PF*_/*S*_*PF*_) and *N*_*S*_ (=*S*_*m-S*_/*S*_*S*_), are shown in Fig. 6. Since the values of *N*_*S*_ are closer to unity than those of *N*_*PF*_, the reverse current is dominated by the Schottky emission.

## Conclusions

We have investigated the electrical characteristics of Mo/Au Schottky barrier diodes fabricated on the (100) β-Ga_2_O_3_ film mechanically exfoliated from the Sn-doped β-Ga_2_O_3_ substrate. On the basis of TE model, the extracted *ϕ*_*b*_ and *n* increases and decreases with the increasing temperature, respectively. By assuming the Gaussian distribution of inhomogeneous barrier height, the mean barrier height of 1.55 eV and the standard deviation of 0.186 eV were obtained*.* Finally, according to the *ln*(*I*/*T*^2^) and *ln*(*I*/*E*) versus *E*^1/2^ plots, the parameter *N*_*S*_ of Schottky emission is close to unity, illustrating the Schottky emission being the dominant transport mechanism of the reverse current. The breakdown voltage of 300 V with samples in Fluorinert is obtained in Mo/Au Schottky barrier diodes with an average electric field of 3 MV/cm, indicating the great potential of β-Ga_2_O_3_ for power electronics applications.

## Abbreviations

I-V: Current-voltage
Mo: Molybdenum
RTA: Rapid thermal annealing
SBD: Schottky barrier diode
TE: Thermionic emission
